# Increased thyroid stimulating hormone (TSH) as a possible risk factor for atherosclerosis in subclinical hypothyroidism

**DOI:** 10.1186/s13044-024-00199-3

**Published:** 2024-06-17

**Authors:** Basil Mohammed Alomair, Hayder M. Al-Kuraishy, Ali I. Al-Gareeb, Majed Ayed Alshammari, Athanasios Alexiou, Marios Papadakis, Hebatallah M. Saad, Gaber El-Saber Batiha

**Affiliations:** 1 Assistant Professor, Internal Medicine and Endocrinology, Department of Medicine, College of Medicine, Jouf University, Sakakah, 04631 Kingdom of Saudi Arabia; 2 Department of Clinical Pharmacology and Medicine, College of Medicine, Mustansiriyah University, Baghdad, Iraq; 3 Department of Medicine, Prince Mohammed Bin Abdulaziz Medical City, Al Jouf-Sakkaka, 42421 Saudi Arabia; 4 University Centre for Research & Development, Chandigarh University, Chandigarh-Ludhiana Highway, Mohali, Punjab India; 5 Department of Research & Development, Funogen, Athens, Greece; 6 Department of Research & Development, AFNP Med, Vienna, 1030 Austria; 7 Department of Science and Engineering, Novel Global Community Educational Foundation, Hebersham, 2770 NSW Australia; 8 Department of Surgery II, University Hospital Witten-Herdecke, University of Witten-Herdecke, Heusnerstrasse 40, Wuppertal, 42283 Germany; 9 Department of Pathology, Faculty of Veterinary Medicine, Matrouh University, Marsa Matruh, 51744 Egypt; 10 Department of Pharmacology and Therapeutics, Faculty of Veterinary Medicine, Damanhour University, Damanhour, 22511 AlBeheira Egypt

**Keywords:** Primary hypothyroidism, Atherosclerosis, Thyroid stimulating hormone

## Abstract

Primary hypothyroidism (PHT) is associated with an increased risk for the development of atherosclerosis (AS) and other cardiovascular disorders. PHT induces atherosclerosis (AS) through the induction of endothelial dysfunction, and insulin resistance (IR). PHT promotes vasoconstriction and the development of hypertension. However, patients with subclinical PHT with normal thyroid hormones (THs) are also at risk for cardiovascular complications. In subclinical PHT, increasing thyroid stimulating hormone (TSH) levels could be one of the causative factors intricate in the progression of cardiovascular complications including AS. Nevertheless, the mechanistic role of PHT in AS has not been fully clarified in relation to increased TSH. Therefore, in this review, we discuss the association between increased TSH and AS, and how increased TSH may be involved in the pathogenesis of AS. In addition, we also discuss how L-thyroxine treatment affects the development of AS.

## Introduction

Atherosclerosis (AS) is a disease of medium and large-size arteries characterized by fatty deposition in the inner part, focal thickening of the arterial wall, and formation of atherosclerotic plaques [1]. The underlying pathological conditions linked with AS progression are inflammation, oxidative stress, endothelial dysfunction, apoptosis, vascular proliferation, matrix degeneration, and neovascularization [2–4]. AS is a progressive disease that interferes with blood flow leading to tissue ischemia mainly in the brain and heart causing stroke and ischemic heart disease respectively [5]. AS affects large and small blood vessels such as femoral and coronary arteries [6]. AS complications like peripheral vascular disease, stroke and myocardial infarction are the major causes of mortality [7]. Surprisingly, AS process may be started in childhood and manifested clinically in middle age and later life [8]. The rupture of atherosclerotic plaques and associated thromboembolic disorders are the main reasons for cardiovascular complications [9].

Dyslipidemia mainly hypercholesterolemia is regarded as the major inducer for the development of AS, as increasing circulating cholesterol increases endothelial permeability which facilitates entry and deposition of lipid particles in the vascular endothelium [10]. Lipid particles mainly low-density lipoprotein (LDL) in the sub-endothelial space act as chemo-attractants for the monocytes which are converted to foamy macrophages [11]. In addition, oxidized LDL in the sub-endothelial space triggers the expression of scavenger receptors on the macrophages with further accumulation of intracellular cholesterol [12]. These pathological changes promote plaque formation, narrowing of the vascular lumen and the development of AS [11–13]. Atherosclerotic plaques are susceptible for erosion, rupture, and calcification with nodule formation [14]. Higher infiltration of T cells into the atherosclerotic plaque increases its vulnerability for rupture and thrombosis [14].

Moreover, increasing levels of LDL and TG with low HDL are regarded as strong predictors for the development of premature AS [11]. High HDL level is considered a protective factor against the development and progression of AS [15]. Besides, hypertriglyceridemia is considered an independent risk factor for the progression of AS [16]. Likewise, lipoprotein disorders are linked with AS pathophysiology. Increased lipoprotein A is associated with AS development [17, 18]. Notably, the macrophage is the most important immune cell involved in the progression of AS and atherosclerotic complications including erosion and rupture [19]. Normally, immune cells mainly macrophages consume oxidized LDL (ox-LDL) with the production of reactive oxygen species (ROS) [19]. In turn, excessive production of ROS promotes the development of oxidative stress and the progression of plaque instability [20]. Therefore, ox-LDL accelerates the macrophage oxidative stress process with the progression of oxidative stress.

Oxidative stress together with inflammation enhances AS progression in a vicious cycle as inflammation induces oxidative stress and vice versa [19, 20]. Oxidative stress promotes the expression of inflammatory signaling pathways, pro-inflammatory cytokines, and chemokines which in turn enhance ROS generation [20]. NADPH-oxidase is a widely expressed enzyme mainly in vascular smooth muscle involved in ROS generation [21]. Higher expression of NADPH-oxidase is increased by the aging process leading to endothelial dysfunction, vascular inflammation, and mitochondrial and cellular-induced oxidative stress [21, 22]. It has been shown that ox-LDL activates the infiltration of monocytes and the migration of smooth muscle cells [23]. It participates in atherothrombosis through induction of the apoptosis of endothelial cells, plaque erosion, production of tissue factors, and impairment of endogenous anticoagulant pathway [23]. In normal physiological conditions, HDL attenuates the production and the effect of ox-LDL [24]. Furthermore, oxidized HDL (ox-HDL) loses its vasculoprotective effect and is regarded as a pro-inflammatory and proatherogenic factor increasing the risk of AS progression [25]. As well, ox-HDL promotes the progression of atherosclerotic plaque erosion and rupture [26]. Therefore, ox-HDL is regarded as a potential risk factor for the development and progression of AS and atherothrombosis. These observations revealed that AS pathogenesis is a complex process related to dyslipidemia and associated inflammatory disorders and oxidative stress.

It has been reported that primary hypothyroidism (PHT) is associated with increasing AS risk [27]. It is thought to be mainly a consequence of reduction of thyroid hormones (THs). The direct impact of increasing levels of thyroid stimulating hormone (TSH) in the mechanistic role of PHT in AS is not fully elucidated. In this review, we aimed to discuss the relationship between increased TSH in PHT and AS, and how increased TSH may be involved in the pathogenesis of AS.

## Primary hypothyroidism

PHT is an endocrine disorder due to the failure of the thyroid gland to produce sufficient THs to maintain the body's metabolic rate [28]. Overt PHT leads to cold intolerance, poor appetite, weight gain, bradycardia, constipation, and depression [29, 30]. However, subclinical PHT which is characterized by normal TH levels and increased TSH is generally asymptomatic [31]. Subclinical PHT represents a compensated state in which increased TSH maintains normal thyroid function [31]. THs including thyroxine (T4) and triiodothyronine (T3) are required for brain growth and development, therefore reduction of these hormones during fetal life lead to congenital hypothyroidism (CH) [32]. The most common cause of PHT is Hashimoto's thyroiditis [33]. Other causes include iodine deficiency [34], radioactive iodine therapy, thyroidectomy and drugs interferon and amiodarone [35]. Of note, PHT through the reduction of circulating THs levels and increase in TSH level leads to systemic adverse effects including impairment of lipid homeostasis, steatogenic effect, expansion of visceral fat and induction of chronic inflammation [36]. These changes lead to insulin resistance (IR), attenuation of hepatic lipoprotein lipase (LPL) activity, and increase inflammatory and oxidative stress disorders [37].

It has been shown that PHT is commonly associated with the development of systemic disorders through the induction of oxidative stress and inflammation [38]. Besides, oxidative stress and inflammation in different systemic diseases may reduce the function of the thyroid gland leading to PHT [38]. Interestingly, long-term PHT can affect the functions of the CNS and increase the risk for the development of Alzheimer's disease (AD) by triggering inflammation and oxidative stress which play a critical role in the pathogenesis of AD [39].

On the other hand, subclinical PHT is often associated with various cardiovascular disorders, low-grade inflammation and endothelial dysfunction [40]. A case–control study confirmed that low-grade inflammation and endothelial dysfunction are more evident in patients with subclinical PHT compared to healthy controls [40]. A systematic review and meta-analysis showed that inflammatory and oxidative biomarkers C-reactive protein (CRP) and malondialdehyde (MDA) are augmented in patients with subclinical PHT [41]. Moreover, subclinical PHT increases the risk for the development of obesity and metabolic syndrome by increasing visceral fat. In turn, increasing visceral fat in obesity and metabolic syndrome induces alterations in the expression of TH receptors and deiodinase activity leading to the exaggeration of subclinical PHT to the overt PHT through the mechanism related to the status of low-grade inflammation [42]. Of interest, inflammation in subclinical PHT as evidenced by the high lymphocyte-to-monocyte ratio which is an indicator of inflammation, contributes to the induction of vascular injury, endothelial dysfunction, and the development of AS [43]. For example, carotid intima-media thickness (CIMT) is positively correlated with TSH serum levels and lymphocyte-to-monocyte ratio in patients with subclinical PHT [43].

These findings suggest that subclinical PHT has a pathogenic role in the development of vascular and metabolic disorders by increasing inflammation and oxidative stress.

## Thyroid-stimulating hormone

TSH also known as thyrotropin is a glycoprotein hormone released from the anterior pituitary under the effect of thyrotropin-releasing hormone (TRH) from the hypothalamus [44]. TSH stimulates thyroid gland to synthesize and release THs. In turn, THs negatively inhibit the release of TSH and TRH from the pituitary and hypothalamus respectively [44]. Moreover, leptin activates the release of TRH from the hypothalamus; however, somatostatin (SS) and dopamine (DA) inhibit TSH release from anterior pituitary [45]. Opioids and alpha-adrenergic stimulate while glucocorticoids and pro-inflammatory cytokines inhibit TSH release [45, 46]. TSH level is increased during the growth period [44]. Glycoprotein TSH is composed of two subunits, the alpha subunit is identical to that found in follicular stimulating hormone (FSH), luteinizing hormone (LH), and human chorionic gonadotropin (HCG) [47]. However, the beta subunit is specific to TSH that is used to determine the TSH level [47].

Although most laboratories have TSH reference around 0.4–4.5 mIU/L, based on the epidemiological survey, the National Academy of Clinical Biochemistry recommended that the TSH reference range should be 0.4–2.5 mU/L [48, 49]. It has been illustrated that TSH > 2.5mU/L is associated with IR and hyperinsulinemia in healthy Korean women [50]. TSH > 2.5mU/L increases the risk for the development of metabolic syndrome [50]. As well, even in euthyroid subjects, higher levels of TSH may be associated with the development of IR and hyperinsulinemia [50]. Moreover, TSH is also increased in patients with metabolic syndrome due to different causes including THs resistance, leptin resistance, and other neuroendocrine alterations [51]. Findings from different clinical studies observed that TSH is positively correlated with circulating leptin levels, IR, and visceral adiposity [50]. Therefore, monitoring of TSH may be required for dyslipidemia and cardiovascular diseases even in euthyroid patients.

TSH acts on specific receptors called TSHRs which are G-protein coupled 7-transmembrane receptors [52]. TSHRs are chiefly expressed on the thyroid epithelial cells, anterior pituitary, and hypothalamus involved in the regulation of THs release and TSH response [52]. The binding of TSH to TSHRs stimulates cyclic adenosine monophosphate (cAMP) signaling and results in the activation of iodide uptake, synthesis of THs and proliferation of thyroid epithelial cells [53]. Extra-thyroidal expression of TSHRs is also present in different tissues and cells including skin, ovary, immune system, kidney, peripheral blood cells, bone marrow, adipose tissue, bone, and endothelial cells [53]. Drvota et al. [54] confirmed the expression of functional TSHRs in the cardiomyocytes. Systemic expression of TSHRs suggests the role of TSH in different cardiometabolic processes and inflammatory reactions regardless of the thyroid gland. It has been reported that high TSH level is associated with an increased risk of cardiovascular diseases by reducing of insulin sensitivity and lipid metabolism [55, 56]. A cross-sectional study conducted by Wang et al. [57] showed that TSH affects lipid metabolism through THs-dependent and THs-independent mechanisms causing hypercholesterolemia. TSHRs are highly expressed in the vascular smooth muscle cells and are involved in the proliferation of these cells via cAMP-dependent pathways [58]. Bell et al. [59] found that TSHRs promote the development of inflammation through the induction of IL-6 release from cultured adipocytes. TSHRs in the vascular endothelium have proangiogenic effects in human microvascular endothelial cell lines by increasing cyclic adenosine monophosphate (cAMP) and vascular endothelial growth factor (VEGF) [60]. Moreover, increasing the expression of TSHRs in the vascular endothelium is involved in the progression of endothelial dysfunction through initiation the expression of Aκt/eNOS [61]. Excessive stimulation of endothelial TSHRs by increased TSH level in subclinical PHT promotes the generation of free radicals and the development of oxidative stress with induction of endothelial dysfunction [61]. Interestingly, TSHRs crosstalk with insulin growth factor 1 (IGF-1), and activation of TSHRs triggers the stimulation of IGF-1 receptor in Graves’ ophthalmopathy [62]. However, IGF-1 has a vasculoprotective effect by inhibiting vascular inflammation and oxidative stress [63]. Thus, the crosstalk between IGF-1 and TSHR seems to be regulatory to prevent TSHR-induced oxidative stress and inflammation. These findings indicated that augmentation of TSH level in subclinical PHT is implicated in the development of endothelial dysfunction which accelerates the development and progression of AS.

## PHT and AS

It is well-known that PHT is associated with the development and progression of AS and other cardiovascular complications such as hypertension and coronary artery disease [64]. PHT induces the progression of AS by increasing LDL, endothelial dysfunction, induction of IR and diastolic hypertension [64]. Increasing levels of LDL, total cholesterol and apolipoprotein B are often present in overt PHT [65]. Of interest, overt PHT is linked with the prolongation of LDL half-life due to reduction of cholesterol catabolism [66]. In addition, PHT induces more detrimental effects on the lipid profile by reducing of HDL synthesis, delaying chylomicron clearance, decreasing the activity of cholesterol and inhibition of hepatic and lipoprotein lipase [67]. In addition, decreasing THs in PHT triggers vasoconstriction and the development of hypertension due to decrease of endothelial nitric oxide (NO) and augmentation of angiotensin II level according to the cross-sectional study and other studies which involved patients with PHT [68, 69]. L-thyroxine replacement in patients with hypothyroidism reverses most of the cardiovascular complications [70]. These findings suggest that THs are the causal factors involved in cardiometabolic disorders in patients with overt PHT. However, patients with subclinical PHT are at risk for cardiovascular complications [71]. In subclinical PHT, higher TSH levels could be the major causative factor in the progression of cardiovascular complications [71]. Subclinical PHT has been associated with an increased incidence of cardiovascular risk factors including AS and dyslipidemia [71]. TSH level > 10 mU/L seems to be a noteworthy predictor of cardiovascular complications [71]. Evidence from different studies confirmed the association between subclinical PHT and cardiovascular diseases [72, 73]. The observational study included women with subclinical PHT showed a higher incidence of myocardial infarction [74]. A study conducted by Lindeman et al. [75] revealed that patients with subclinical PHT with TSH levels > 10 mlU/L were associated with coronary heart disease compared with those with TSH levels < 4.6 mlU/L. A longitudinal study showed a positive correlation between cardiovascular events and subclinical PHT [76]. Notably, subclinical PHT exacerbates cardiovascular risk and cardiac deaths in patients with underlying cardiovascular diseases compared with euthyroid patients [77]. In this state, subclinical PHT is associated with higher mortality in patients with cardiovascular diseases [78]. However, other studies showed no positive correlation between subclinical PHT and risk of cardiovascular disorders [79]. Many meta-analyses showed that subclinical PHT was associated with cardiovascular complications and mortality [80, 81]. However, a cohort study involved 344 patients with angina pectoris exhibited that TSH level did not predict the severity of coronary artery diseases in euthyroid patients [27]. Therefore, L-thyroxine replacement therapy improves lipid profile and prevents the proatherogenic process [82]. It has been observed that subclinical PHT is regarded as a potential risk factor for the development of AS. A cohort study involved 100 women with subclinical PHT, 45 women with overt PHT and 42 healthy women as controls. This study observed that subclinical PHT and associated dyslipidemia increase the risk for the development of AS, though treatment with L-thyroxine reduced AS risk within six months [82]. Low THs and increased TSH level affect many target organs by changing their morphology and function and by inducing the development of AS. The increased risk of acceleration and extension of AS in patients with PHT could be explained by dyslipidemia, diastolic hypertension, increased arterial stiffness, endothelial dysfunction, and altered blood coagulation [83]. Instability of AS plaque in PHT could activate innate immunity, which is involved in the formation of AS plaques. Optimal L-thyroxine replacement therapy restores biochemical euthyroidism. In postmenopausal women and elderly patients with PHT and associated vascular comorbidity, excessive LT4 substitution could lead to atrial rhythm disorders and osteoporosis [83]. Therefore, it is of interest to maintain TSH levels in the reference range, thus eliminating the harmful effects of lower or higher TSH levels on the cardiovascular system. A systematic review and meta-analysis involving 26 clinical studies of 36,434 patients with thyroid dysfunction indicated that L-thyroxine replacement therapy in patients with PHT reduces the risk of AS after one year [84] suggesting that high TSH and dysregulated THs in PHT are intricate in the proatherogenic process. As well, pulse wave velocity which is an indicator of arterial stiffness and AS is augmented in patients with subclinical PHT compared to healthy controls [85]. Besides, TSH level is positively correlated with pulse wave velocity in patients with subclinical PHT [85]. A prospective study on 62 patients with subclinical PHT and 64 healthy controls revealed that the level of vasculoprotective dehydroepiandrosterone sulfate (DHEA-S) was reduced in patients with subclinical PHT compared to the healthy controls [86]. Supporting this claim, a previous experimental study observed that low DHEA-S was associated with increasing risk of AS [87].

Together, both hypertension and dyslipidemia that develop in PHT promote the development of oxidative stress and inflammatory disorders leading to the development of AS. These findings implicate PHT in the pathogenesis of AS and cardiovascular complications through various mechanisms including inflammation and oxidative stress in both overt and subclinical states.

## TSH in the pathogenesis of AS

### TSH and inflammation

It has been shown that TSH plays a critical role in the pathogenesis of AS through different pathways. A cohort study included 744 women with normal thyroid function according to normal TSH levels (0.3–4.9µU/ml), women with TSH levels > 2.1 µU/ml had a higher risk for the development of cardiovascular disorders due to AS [88]. A case–control study including 69 patients with subclinical PHT and 30 matched healthy controls revealed that TSH level is correlated with high CIMT which is a marker of carotid AS [89]. This finding indicated that the development of AS is a consequence of subclinical PHT, and a high TSH level in subclinical PHT is regarded as an independent risk factor for the development of AS. An experimental study conducted by Yang et al. [90] revealed that ablation of TSHRs in *ApoE*^−/−^ mice attenuates the development and progression of AS by inhibiting macrophage activity and associated inflammation in atherosclerotic plaque. Also, in vitro study demonstrated that TSHRs promote inflammation, the release of pro-inflammatory cytokines, monocyte recruitment, and macrophage activation via a mitogen-activated protein kinase (MAPK)-dependent mechanism [90]. Therefore, TSH/TSHRs are involved in the pathogenesis of AS through the induction of vascular inflammation. Nevertheless, various studies revealed that the causal relationship between TSH and AS is uncertain [91, 92]. Cooper and Bondi [91] suggest that treatment of subclinical PHT patients with L-thyroxine may not prevent AS. Delitala et al. [92] and other studies elucidated that TSH-induced AS is mediated through the alteration of lipid metabolism and the development of dyslipidemia [93, 94]. However, the correlation between CIMT with TSH remains distorted after the correction of the lipid profile [89] suggesting that TSH may lead to AS by an independent mechanism.

Inflammation is regarded as a cornerstone in AS pathogenesis, as unrestrained inflammation inhibits macrophage cholesterol efflux leading to cholesterol accumulation and the formation of atherosclerotic plaque [95]. In turn, atherosclerotic plaque triggers inflammatory changes by releasing pro-inflammatory cytokines from macrophages in the atherosclerotic plaque causing extensive tissue injury [95, 96]. Furthermore, inflammatory mediators released by plaque macrophages aggravate local tissue damage, which in turn provoke more inflammation in a vicious circle manner. It has been shown that TSH contributes to AS development by inducing the activation of macrophages and the release of inflammatory cytokines [97]. Yang et al. [98] found that TSH has a pro-inflammatory effect on macrophages through the activation of phospholipase C (PLC) and the Rho-GTPase pathway. TSHRs have also been reported to be expressed in macrophages, endothelial cells, and smooth muscle cells. These three types of cells are most critically involved in atherosclerosis. Therefore, TSH can promote atherosclerosis not only indirectly by regulating thyroid function, but also directly by acting on these cells [98]. High TSH inhibits peroxisome proliferator activator receptor gamma (PPAR-γ) which is an imperative molecule in the resolution of inflammation and lipid homeostasis [90]. In addition, TSH attenuates the expression of liver X receptor alpha (LXRα) and ATP-binding cassette A1 (ABCA1) which are involved in cholesterol transport leading to aggravation of cholesterol accumulation in the atherosclerotic plaque [99, 100]. As well, TSH augments macrophage burden by inducing the recruitment of monocytes [97, 99, 100]. Moreover, TSH activates many inflammatory signaling pathways like nuclear factor kappa B (NF-κB), MAPK and node-like receptor pyrine 3 (NLRP3) inflammasome [101]. Of note, THs inhibit the expression of NLRP3 inflammasome and attenuate inflammatory disorders [102]. Thus, subclinical PHT with increasing TSH levels may induce inflammatory changes through the activation of NLRP3 inflammasome [102]. Of note, NF-κB and MAPK collaborate in the expression of macrophage chemokines leading to the recruitment of monocytes [101]. TSH regulates immune function in normal and disease states as in AS [101]. At the physiological level, TSH has immunomodulatory effects, though its pathological level triggers pro-inflammatory conditions. In the early stage of PHT, increasing TSH levels leads to direct pro-inflammatory action, endothelial dysfunction, and induction of AS pathogenesis [74, 97]. Furthermore, accelerated AS in patients with subclinical PHT may be due to the suppression of transforming growth factor-β-activated kinase 1 (TAK1) and AMP-activated protein kinase (AMPK) pathway which reduce the activity of macrophages [103]. Increased TSH level in subclinical PHT inhibits AMPK in the primary mouse hepatocytes and HepG2 cells [104]. TSH suppresses macrophage TAK1 in HepG2 cells [103]. Therefore, inhibition of TAK-AMPK pathway triggers the induction of macrophage and the release of pro-inflammatory cytokines. Thus, TSH-induced vascular inflammation could be the potential mechanism in the development and progression of AS.

### TSH and oxidative stress

Oxidative stress is involved in the pathogenesis of various cardiovascular diseases including AS [20]. AS represents a state of oxidative stress characterized by protein and lipid oxidations in the vascular endothelium [20]. The overproduction of ROS is integral in the development and progression of endothelial dysfunction and AS [105]. Oxidative stress-induced endothelial dysfunction is mediated by the depletion of endothelium NO [106]. It has been shown that oxidative stress promotes the formation of atherosclerotic plaque through the induction the expression of adhesion molecules, inflammation and the development of endothelial dysfunction [107]. Endothelial NADPH oxidase is a master enzyme for the generation of ROS that correlates with the progression of endothelial dysfunction and AS [108]. Different studies revealed that LDL directly activates endothelial NADPH oxidase via the expression of signal transduction like phospholipase A2 and the release of arachidonic acid (AA) which is involved in the activation of NADPH oxidase [109]. Monocytes, macrophages, VSMCs, and endothelial cells can oxidize LDL through NADPH oxidase-dependent pathway [110]. Moreover, ROS are also produced by other enzymes and pathways including xanthine oxidase, mitochondrial eNOS and uncoupling eNOS [111]. The vascular endothelium is protected from the effect of oxidative stress by antioxidant enzyme systems such as catalase, superoxide dismutase, paraoxonase and glutathione peroxidase [112]. ROS induces atherogenesis by oxidative modification of phospholipids and lipoproteins [12]. Therefore, oxidative/antioxidant imbalance promotes macrophage polarization, formation of foam cells and the development of atherosclerotic plaque.

It has been shown that PHT is associated with the development of oxidative stress due to the generation of ROS and reduction of antioxidant enzymes mainly paraoxonase (PON)-1 and superoxide dismutase (SOD) [113]. Subclinical PHT is also linked with the development of oxidative stress [114]. A case–control study included 467 males with subclinical PHT compared to 190 healthy controls illustrated that oxidative stress markers were higher in patients with subclinical PHT compared to controls [114]. A case–control study showed that the activity of antioxidant enzymes such as superoxide dismutase, catalase and arylestrase were reduced in patients with subclinical PHT compared to controls [114]. Oxidative stress biomarker, MDA level is higher in patients with subclinical PHT compared to healthy controls. After treatment with L-thyroxine, the stress marker is reduced to a significant extent. MDA can be used as a useful biomarker to measure and monitor oxidative stress in PHT [115]. Therefore, the underlying cause for the development of oxidative stress in PHT and subclinical PHT is due to reduction of thyroid function. However, the fundamental role of TSH in the development of oxidative stress was suggested [116]. Fan et al. [116] illustrated that TSH aggravates peripheral neuropathy in diabetic patients through augmentation of oxidative stress. Stimulatory autoantibodies for TSHRs in Graves' disease promote oxidative stress [117] supporting that TSH is the main cause for the progression of oxidative stress in PHT. It has been shown that TSH is directly involved in the development of oxidative stress by inducing lipid and protein peroxidation in patients with subclinical PHT by inducing the generation of ROS [118]. In addition, increased TSH in subclinical PHT activates dual oxidase and NADPH oxidase enzymes resulting in the excessive production of ROS [119]. Furthermore, the antioxidant defense mechanism is impaired in subclinical PHT due to the augmentation of lipid peroxidation [113]. Increased TSH level in subclinical PHT inhibits the functional activity of antioxidant enzymes [119]. Consequently, TSH through induction of oxidative stress increases the risk for the development of AS and other cardiovascular complications in subclinical PHT.

### TSH and dyslipidemia

Dyslipidemia is a disorder of plasma protein characterized by an increase of serum cholesterol, triglyceride, LDL, and VLDL and reduction of beneficial HDL [113, 120, 121]. It has been shown that dyslipidemia disrupts the endothelial function by inhibiting the release of NO through increment of endogenous NO inhibitor asymmetric dimethylarginine (ADAM). Dyslipidemia inhibits the activity of dimethylarginine dimethylaminohydrolase (DDAH) [122]. However, HDL improves the release of NO from vascular endothelium through modulation of endothelial Ca^+2^ releases [122]. As well, LDL-induced endothelial dysfunction is implicated in the pathogenesis of AS [123]. Furthermore, subclinical PHT can trigger the development of endothelial dysfunction through the induction of dyslipidemia [124]. It has been illustrated that increasing TSH level in subclinical PHT is positively correlated with serum triglyceride, cholesterol, and LDL, and negatively correlated with serum HDL level [125]. Also, a high TSH level may increase the development of dyslipidemia through induction the development of IR [126]. Backup to these findings, mutations of TH receptor β induce peripheral resistance to the effect of THs which leads to the development of dyslipidemia [127]. A large cross-sectional study revealed that TSH is positively correlated with serum triglyceride [128]. The mechanism for the development of dyslipidemia in subclinical PHT is through the activation of hepatic HMG-CoA a rate-limiting enzyme by TSH [129]. TSH through activation of the hepatocyte TSHR which induces activation of the cAMP/PKA/CREB signaling pathway leads to the development of dyslipidemia [129]. Also, TSH activates sterol regulatory element-binding protein 2 (SREBP2), a major transcription factor, and SREBP2 can stimulate the transcription of the *HMGCR* gene [130]. Moreover, THs increase the expression of LDL receptors which eliminate circulating LDL [131] therefore; in PHT the clearance of LDL is reduced leading to hypercholesterolemia. Interestingly, increasing TSH in subclinical and overt PHT induces lipolysis. An in vitro study showed that injection of TSH significantly increases free fatty acid by activating lipolysis [132]. In addition, TSH stimulates the expression of ApoB leading to increasing synthesis of plasma lipids [132]. Likewise, augmentation of TSH serum levels inhibits the clearance of LDL through bile [133]. In patients with subclinical PHT, TSH is negatively correlated with the levels of bile acid independent of TH level [134]. Therefore, increasing TSH levels in subclinical PHT promotes the development and progression of AS through the induction of dyslipidemia which is the main contributing factor in the pathogenesis of AS.

### Cardiometabolic portfolio of TSH

Furthermore, pathological TSH level contributes to various cardiometabolic disorders including hypertension [135], IR [136] and thrombosis [137]. These cardiometabolic disorders are connected with the development and progression of AS. Increasing high-sensitive TSH levels is correlated with hypertension, fibrinogen, IR, dyslipidemia, inflammation, hypercoagulability, and metabolic risk factors in the Taiwanese population [135]. Additionally, TSH promotes the development of IR by increasing visceral adipose tissue [136]. However, Stocia and colleagues found no association between subclinical PHT and IR in adult Romanian women in a retrospective study [138]. Of interest, IR induces dyslipidemia, endothelial dysfunction, and alteration of insulin signaling that promotes the development of atherosclerotic plaque [139]. During IR, chronic hyperglycemia and related oxidative stress trigger inflammatory responses leading to the development of endothelial dysfunction and plaque formation [139]. It has been illustrated that TSH level is correlated with IR. The mechanism of the development of IR in subclinical PHT is related to the downregulation of peripheral insulin receptors and increase in the release of prolactin which is activated by high TRH [140]. Together, increased TSH, IR and hyperinsulinemia promote the development and progression of AS. In patients with subclinical PHT, TSH raises the risk of thrombosis; hence, anti-thrombotic medications are advised in PHT management [137]. It has been observed that hypertension increases AS by inducing endothelial dysfunction [141]. Therefore, TSH through induction of hypertension, IR and thrombosis leads to induction of AS. The underlying causes for TSH-induced cardiometabolic disorders may be through augmentation of oxidative and stress disorders which per se lead to AS development.

### TSH and autophagy

Autophagy is a specific subcellular process involved in the degradation and recycling of damaged organelles and proteins to maintain cellular homeostasis [142]. Dysregulation of autophagy is associated with the development of many cardiovascular disorders including AS [142]. The defective autophagic process in the endothelial cells promotes apoptosis and the development of AS [142, 143]. Macrophage autophagy is an anti-atherogenic process through maintains cellular lipid homeostasis by increasing cytosolic lipid catabolism [143]. Improvement of this process may reduce the risk of AS [143]. An experimental study demonstrated that metformin attenuates high-fat diet (HFD)-mediated AS and maintains plaque stability by enhancing the macrophage autophagic process [143]. Of note, autophagy plays a critical role in various thyroid diseases or in various phases of the same thyroid disease [144]. In PHT, the autophagic process is severely impaired through an IL-1-mediated pathway [145]. A previous study observed that THs improve lipid metabolism through the induction of autophagy [146]. Blocking of the autophagic process by autophagy-related 5 siRNA reduces THs-mediated lipid metabolism by β-oxidation [146]. However, TSH inhibits autophagy and promotes apoptosis [147]. Kurashige et al. [148] illustrated in vitro study that TSH regulates the autophagic process as the metabolites produced by autophagy promote protein synthesis. Conversely, different studies revealed that TSH activates autophagy [149, 150].

These findings showed that TSH promotes autophagy which could be protective rather than detrimental in AS. The autophagic process is critical in the pathogenesis of AS, as it is protective by preventing the accumulation of lipids in the lipid core with the atherosclerotic plaque, though excessive autophagy may induce rupture of the atherosclerotic plaque [151]. Notoriously, the autophagic process may induce a detrimental effect on AS pathogenesis by reducing collagen synthesis with decreasing plaque stability [152]. The exaggerated autophagic process impairs endothelial functions causing endothelial apoptosis and regional thrombosis [152]. Autophagy-induced cell death is provoked by autophagosomes through uncontrolled lysosomal dysfunction [153]. Markedly, an experimental study showed that enhanced autophagy may increase the development and progression of AS [154]. These findings implicate the role of autophagy in AS progression. Therefore, TSH-induced autophagy may exacerbate AS progression and related complications by inducing the destability and rupture of the atherosclerotic plaque.

### TSH and plaque stability

It has been shown that macrophages within atherosclerotic plaque are regarded as the major source of pro-inflammatory and inflammatory cytokines [155]. Macrophage is considered as a key regulator of metabolic signals and inflammatory response in atherosclerotic plaque formation. Therefore, macrophage activity and plaque contents are changed in a dynamic balance [19]. Macrophage lipid contents trigger inflammation and immune response by augmentation of the sensitivity of TLR-4 to their ligands by inducing the expression of NLRP3 inflammasome [156]. The interaction of ox-LDL with monocytes/macrophages in the atherosclerotic plaque promotes inflammatory and oxidative stress disorders [157].

It has been shown that TSH increases CIMT independent of AS risk factors [89]. A systematic review including 26 studies showed that high TSH in subclinical PHT is associated with increasing AS risk and the formation of atherosclerotic plaque [84]. However, TSH-induced inflammatory and oxidative stress disorders together with the acceleration of autophagy promote AS complications by inducing instability and rupture of the atherosclerotic plaque [152]. High TSH level in subclinical PHT triggers abnormal immune response through excessive innate immune response and oxidative stress within the atherosclerotic plaque [83]. TSH activates the expression of matrix metalloproteinases (MMPs) which promotes the degradation of plaque collagen in the atherosclerotic plaque [83]. These changes provoke erosion and rupture of atherosclerotic plaque with subsequent progression of AS complications. Circulating MMPs levels are increased in rats with PHT leading to diffuse tissue injury and endothelial dysfunction [158]. Thus, the fundamental mechanism for rupture of atherosclerotic plaques in patients with AS is related to the augmentation of autophagy and MMP which induced by TSH [83, 152]. These verdicts suggest that TSH in subclinical PHT is regarded as an independent risk factor for the rupture of atherosclerotic plaque in AS.

### TSH and homocysteinemia

Homocysteine is a sulphur and thio-containing amino acid produced by methionine demethylation through methionine demethylase [159]. Homocysteine is involved in the metabolism of methionine and cysteine [159]. Homocysteine is chiefly derived from methionine present in the food, but it does not participate in protein synthesis as it is a non-proteinogenic α-amino acid [159]. About 80% of plasma homocysteine is bound to albumin, though some portions remain free or bound to cysteine to form homocysteine-cysteine disulfide [160]. Homocysteine plasma level is about 10-20µmol/L which is higher in men than women[161]. Homocysteine plasma levels of more than 15µmol/L is called hyperhomocysteinemia which is associated with aging and deficiency of folate, B6 and B12 [161]. Homocysteine level 15–30 µmol/L is regarded as mild hyperhomocysteinemia, 30–100 µmol/L is moderate and more than 100 µmol/L is considered as severe hyperhomocysteinemia. Homocysteine in the plasma undergoes three changes including remethylation to form methionine, trans-sulfation with serine and released to extracellular fluids [162]. The causes of hyperhomocysteinemia could be genetic like congenital hyperhomocysteinemia due to methionine synthase deficiency, or nutritional deficiencies like folate, B6 and B12 [163]. Age is regarded as an important risk factor for the development of hyperhomocysteinemia [164]. Plasma homocysteine progressively increased with increasing age [164]. The positive correlation between homocysteine level and age could be due to deficiency in folate, B6 and B12, kidney impairment, and reduction in the activity of enzymes that are involved in the elimination of homocysteine [164].

It has been reported that TSH level is positively correlated with homocysteine serum levels in patients with PHT [163, 164]. An observational study involved 64 PHT patients compared to 64 healthy controls showed that TSH level in PHT patients was correlated with lipid profile and homocysteine serum level significantly compared to the controls [165]. Hyperhomocysteinemia in PHT is developed due to impairment the metabolism and clearance of homocysteine [165]. As well, IR in subclinical PHT contributes to the development of hyperhomocysteinemia by reducing of homocysteine metabolism [128]. Sengul et al. [166] disclosed that homocysteine serum levels in patients with subclinical PHT was higher compared to controls. Of interest, L-thyroxine replacement therapy improves hyperhomocysteinemia in patients with subclinical PHT through the improvement of homocysteine metabolism [84]. Hyperhomocysteinemia in PHT promotes the development of IR and cardiovascular complications [167]. Hyperhomocysteinemia is regarded as a potential risk factor for the development and progression of AS [168]. Hyperhomocysteinemia is an independent risk factor for AS through the induction of endothelial dysfunction, vascular inflammation, and release of pro-inflammatory cytokines [169]. These findings pointed out that TSH-induced hyperhomocysteinemia might be a possible mechanism in the development of AS.

**Fig. 1:**
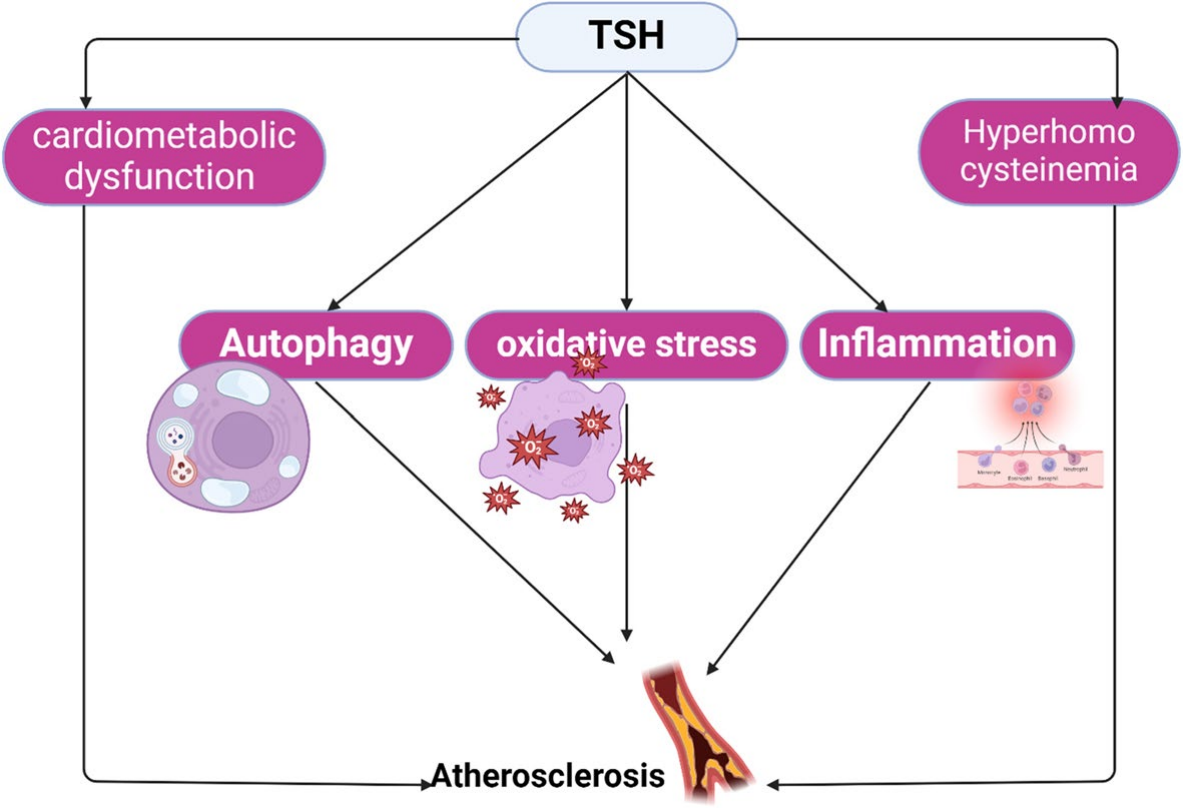
Hypothyroidism-accelerated atherosclerosis

Taken together, TSH-mediated AS could be related to the development of vascular inflammation, oxidative stress, induction of autophagy, hyperhomocysteinemia, and acceleration of cardiometabolic dysfunction including IR and hypertension. Subclinical PHT with high TSH leads to hypertension, IR, hypercholesterolemia, hyperhomocysteinemia, and vascular complications (Fig. 1).

## L-thyroxine and AS

L-thyroxine is mainly used as a replacement therapy in the management of PHT. However, appropriate use of L-thyroxine may be indicated in subclinical PHT to restore normal TH levels and to reduce TSH levels by a negative feedback mechanism. It has been shown that 6-month treatment with L-thyroxine improves endothelial dysfunction and prevents atherogenesis in patients with subclinical PHT [170]. Likewise, different clinical trials observed that treatment with L-thyroxine improves endothelial dysfunction, lipid profile and CIMT in patients with subclinical PHT [171, 172]. However, different studies confirmed that L-thyroxine treatment is insufficient to reduce metabolic complications in patients with subclinical PHT [172–174]. In addition, L-thyroxine treatment is inadequate to restore CIMT in patients with subclinical PHT [174]. Of interest, the treatment of patients with subclinical PHT with L-thyroxine reduces the risk of ischemic heart diseases in the younger but not older group [175].

Concerning the role of L-thyroxine treatment in relation to the risk of AS, it has been shown that L-thyroxine treatment in patients with subclinical PHT reduces the early biomarkers of AS [176]. L-thyroxine treatment inhibits dyslipidemia and reduces ADMA expression and homocysteine levels in patients with subclinical PHT [176]. Alibaz et al. [177] found that L-thyroxine treatment improves endothelial function measured by FMD in patients with subclinical PHT compared to healthy controls. However, a prospective study showed that L-thyroxine treatment improves only the blood pressure and did not ameliorate glucose homeostasis, systemic inflammation, dyslipidemia and coagulation profile in patients with subclinical PHT compared to healthy controls [178]. This study with small sample size did not measure the sequential levels of lipid profile and other parameters regarding the different doses of L-thyroxine in patients with subclinical PHT. Of interest, a prospective study showed that 3 months of L-thyroxine treatment in patients with subclinical PHT ameliorates most of the biomarkers of AS compared to the controls [179]. These findings highlighted that L-thyroxine treatment could be an effective preventive measure against the development of AS through modulation of lipid profile, coagulation and inflammatory biomarkers. Remarkably, cardiovascular disorders such as AS are reversible at euthyroid status, and early diagnosis of subclinical PHT, and treatment with L-thyroxine may prevent the development of cardiovascular complications [180]. However, a case–control study observed that L-thyroxine treatment may aggravate ventricular dysfunction and pulmonary artery stiffness in patients with subclinical PHT [181]. Therefore, careful monitoring of L-thyroxine treatment is advisable mainly in patients with subclinical PHT and associated cardiac dysfunction. The underlying mechanism for the protective effect of L-thyroxine against AS development in patients with subclinical PHT is related to the direct effect of L-thyroxine on the metabolic profile or indirect effect by restoration of normal TSH which implicated in the pathogenesis of AS.

## Conclusion

In subclinical PHT, increased TSH levels may be implicated in the progression of cardiovascular complications. Subclinical PHT has been shown to be associated with increased incidence of cardiovascular risk factors including AS. TSH-induced AS might be due to the development of vascular inflammation, oxidative stress, induction of autophagy, hyperhomocysteinemia, and acceleration of cardiometabolic dysfunction such as IR and hypertension. These findings suggest that TSH is regarded as an independent risk factor for the development and progression of AS. Therefore, L-thyroxine treatment could be an effective preventive measure against the development of AS through modulation of lipid profile, coagulation and inflammatory biomarkers. The underlying mechanism for the protective effect of L-thyroxine against AS development in patients with subclinical PHT is related to the direct effect of L-thyroxine on the metabolic profile or indirect effect by restoration of normal TSH which is implicated in the pathogenesis of AS.

## Data Availability

Not applicable.

## Abbreviations

AS: Atherosclerosis
LDL: Low-density lipoprotein
ROS: Reactive oxygen species
TSH: Thyroid stimulating hormone
THs: Thyroid hormones
T4: Thyroxine
T3: Triiodothyronine
CH: Congenital hypothyroidism
IR: Insulin resistance
LPL: Hepatic lipoprotein lipase
SS: Somatostatin
DA: Dopamine
FSH: Follicular stimulating hormone
LH: Luteinizing hormone
HCG: Human chorionic gonadotropin
cAMP: Cyclic adenosine monophosphate
IR: Insulin resistance
NO: Nitric oxide
CIMT: Carotid intima-media thickness
MAPK: Mitogen-activated protein kinase
PLC: Phospholipase C
PPAR-γ: Peroxisome proliferator activator receptor gamma
LXRα: Liver X receptor alpha
ABCA1: ATP-binding cassette A1
NLRP3: Node like receptor pyrine 3
AA: Arachidonic acid
PON: Paraoxonase-1
SOD: Superoxide dismutase
MMPs: Matrix metalloproteinases
HMGCR: 3-Hydroxy-3-methylglutaryl coenzyme A reductase
PKC: Protein kinase c
